# Reviewing the Role of Instagram in Education: Can a Photo Sharing Application Deliver Benefits to Medical and Dental Anatomy Education?

**DOI:** 10.1007/s40670-019-00767-5

**Published:** 2019-07-16

**Authors:** Naomi Katherine May Douglas, Max Scholz, Matthew Alex Myers, Shivani Margaret Rae, Ahmad Elmansouri, Samuel Hall, Scott Border

**Affiliations:** 1 Department of Dentistry, University of Bristol, Bristol, UK; 2 Department of Engineering, Brunel University London, London, UK; 3 Centre for Learning Anatomical Sciences, University of Southampton, Southampton, UK; 4 Centre for Learning Anatomical Sciences, Faculty of Medicine, Southampton General Hospital, Mailpoint 845, Tremona Road, Southampton, SO16 6YD UK

**Keywords:** Instagram, Education, Anatomy, Dentistry, Teaching

## Abstract

Instagram is an increasingly popular social media site tailored towards sharing photos and videos. An audit investigating current Instagram accounts focusing on anatomy education found a variety of successful teaching styles, including clinical images, descriptive videos, multiple-choice questions, and cartoons. Utilising Instagram for educational purposes, benefits such as ease of use, hashtags, and its effectiveness in conveying visual topics should be weighed against limitations such as passive learning and the requirement of committed staff to oversee its use.

## Introduction

When teaching subjects like anatomy, that involve a considerable ability to visually identify structures, the incorporation of social media has become a valuable resource. As the number of hours dedicated to anatomy teaching in UK Universities decreases, the application of social media tools could become increasingly important [1].

Reports on the use of social media sites used in anatomy education include Facebook, Twitter, and YouTube. In the third quarter of 2017, Facebook had on average 1.37 billion daily users, making it the most popular form of social media [2]. In fact, a 2011 study found that 90% of medical students used Facebook, a figure which has likely grown since [3]. Using Facebook in medical education has been found to encourage collaboration and provide quick feedback because of its ease of use. It is also well tailored in sharing media from other sites like Instagram and YouTube [4]. However, some pitfalls do exist which are mainly focussed around distraction, professionalism, privacy, and safety [4].

Twitter is another notable media site used professionally by academics. In a study looking into the use of a Twitter hashtag for neuroanatomy teaching, it was found that it could be used to boost the morale of students and encourage revision. However, it required regular engagement from the teachers, and there was no evidence to suggest that it had a direct impact on their grades [5].

YouTube has become a particularly successful tool in anatomy teaching for both medical and dental students with greater potential [6, 7]. However, drawing parallels with Twitter, the study did not find that its use boosted exam grades, although it did encourage motivation of learning and enthusiasm. This finding is supported by Barry et al. who reported that 78% of medical students used YouTube as their main source for educational anatomy videos, demonstrating its value in medical teaching [1].

Despite this, there are some recognised limitations to using YouTube as an educational tool. These include criticisms by some educators that learning through watching a video is a passive experience, without the need for student interaction. To overcome this pitfall, educators are developing screencasts—a real-time screen recording application, which can promote engagement by encouraging students to write/draw along with a tutorial. These can be used to supplement teaching and used with a flipped classroom approach to teaching and there is some evidence to suggest that they can work as well as traditional learning resources [8].

In the current climate, a decline in engagement between students and social media has recently been observed, most notably on Twitter and Facebook; however, there has also been a noticeable increase in students shifting to Instagram as their preferred social media platform for educational content [9]. As a result of the changing landscape of social media use in medical education, the purpose of this article is to review the role of Instagram in medical and dental education—with a special focus on clinical anatomy, including its benefits and limitations.

### Emergence of Instagram as an Educational Resource in Health Education

Instagram is a photo- and video-sharing social networking service owned by Facebook, Inc. and was launched in October 2010. The main feature of the application allows users to upload photos (and 60-second videos), which can be edited with various filters and organised with tags and comments (using the hashtag ‘#’ symbol). Posts can be shared publicly or to followers; users can browse other users’ content by tags and locations, and view trending content. Users can also ‘like’ photos and follow other users to add their content to a feed. Focussing mainly on image uploads, Instagram has become the most popular photo sharing platform on the internet. Evidence suggests that it attracts the younger generation with 90% of its 150 million users under the age of 35. In 2017, Instagram reached 800 million users according to its press website. The proportion of these users who are students is unknown, however, a 2016 survey showed that 59% of internet users who use Instagram are between the ages of 18 and 29 [10].

As with other social media sites, Instagram is utilised predominantly for recreational use. Despite this, an increasing number of anatomy education-related Instagram accounts for both medical and dental students are emerging and becoming well-known. Current research includes an investigation into the use of Instagram accounts which post photos and videos related to plastic surgery [11]. The manuscript highlights the visual nature of Instagram and how it suits surgical content. Another study of 234 pharmacy students found that students enjoyed Instagram, but there was no direct evidence of an improvement to examination scores [12].

The defining characteristic of Instagram is mobile image uploads. This aligns very well to anatomy education since the subject benefits greatly from visual aids to support understanding and interpretation [13]. It also allows for an insight and integration into related practices that students may not usually experience. For example, the account ‘seattlesciencefoundation’ posts videos of human dissection from a Dissecting Room, educating the public on the process behind cadaver preparation, whilst also encouraging anatomical interpretation.

There are, of course, some limitations; the main one being a lack of quality control, although this is not a drawback exclusive to the use of Instagram. Anybody can post material within the terms and conditions of the site which can easily result in erroneous information being shared, particularly amongst non-experts. It also introduces concerns regarding the ethical and legal policies of posting of sensitive or personal content. Lack of quality control means that the uploader is responsible for de-identification of individuals and safeguarding of information. Instagram has its own terms of use, which include banning unlawful and confidential content, but it relies on other users being aware of the terms and reporting it, by which time it will have already been posted [14]

## Anatomy Education on Instagram: Search Strategies and Findings

At the present time, little is known about the exact nature of education accounts and their providers; therefore, we conducted an audit based enquiry into Instagram accounts using the key search terms ‘anatomy’, ‘anatomy education’, ‘medicine’, and ‘dentistry’. The most popular and relevant accounts were listed first in this search and the first 5 to 10 profiles were recorded per search. On occasion, related accounts would be recommended by Instagram and the data from these were also included. Profiles were excluded if the content was not predominantly medicine or dentistry based.

Several aspects of account information were collected and recorded in the table below (Table 1) to gain an understanding of the type of accounts that exist, the type of content posted and the size of their audience, including its growth from January 2018 to June 2018.

**Table 1:** A summary of the medical/dental anatomy-related Instagram, including the content, account owner, number of followers, and number of posts. The keywords used to search for the Instagram accounts were as follows: Anatomy; Suggestions from the seattlesciencefoundation; Anatomy education; Medicine; Dentistry; Education; SBH follows; Medical education; SarahJClifford follows; Accounts recommended by Instagram

Search code	Page title	Date of first post	No. of posts	Country of origin	Discipline/subject area	Content type	Followers January 2018	Followers June 2018	Profit making/commercial?	Institutional/individual	Staff/student
1	guided.dental	13/08/2017	42		Dental Education - oral surgery, anatomy, perio, pros/restorative, ortho, endo, implantology	Digitally produced information sheets including educational questions	12,900	21,900		Institutional	Staff
1	seattlesciencefoundation	09/07/2015	482	USA	Medical Research and Education	Surgery and dissection videos	84,000	93,900		Institutional	Staff
1	oralmaxillofacial (Educational OMFS)	30/09/2015	55		Oral and Maxillofacial Surgery	Traumatic head injury photographs, maxfax photographs, teeth radiographs	1008	1133		Unsure	Unsure
2	neurosurgeryresident	28/11/2014	247		Neurosurgery	Photos of neurosurgery cases, brain radiographs,	42,000	55,000		Community?	Staff
2	medical.doctors (Medical Doctors Worldwide)	11/09/2015	1544	Worldwide	Medicine and Health	Medical news, information, motivation, educational pictures and articles	509,000	551,000		Individual	Staff and Students
2	medicalschoolposts	03/09/2014	1005	Worldwide	Medicine and Health	Photographs of unique medical cases, uplifting photographs of patients and family	372,000	Gone		Community?	Staff
2	telehealther	18/08/2016	462		Education Website, smart health trackers, blood pressure monitors, stethoscope sales	Surgery and dental reposted educational videos, health product advertisements	320,000	296		Commercial	Institutional
2	medicalcortex	20/02/2016	1006		Medical education, jewellery sales,	Medical case videos, memes, jewellery advertisements, educational photos and videos	164,000	185,000	Commercial		
2	medschoolstudent (Med School Posts)	15/04/2016	190		Medical Education and humour	Educational posters, memes, and jokes	146,000	195,000		Individual	Student
2	med_students0	01/03/2015	1357		Medical and Health	Surgical videos and animated surgical techniques	165,000	185,000		Individual	Student
2	anatomia.repost	27/06/2016	1745		Anatomy	Surgical videos, dissection specimens, guides to anatomy, rare cases	255,000	311,000			YouTube account
3	aloeanatomy	23/04/2017	28		Musculoskeletal Anatomy	Information on bone structure and positioning, nerves, muscles		673	3133	Commercial	Institutional
4	medicine_on_indexcards	02/09/2017	20		England	Physicians Associate Anatomy, Pathology	Index cards with medical illustrations on them, prize giveaways	1156	1615	Individual	Student
4	medicine_in_pictures	23/09/2016	670		Medicine and Health	Informational diagrams, memes, surgical images, adverts	61,600	72,800	Commercial	Institutional	
4	surgerypics (High Definition Medicine)	24/06/2013	339		Medical Education	Re-posted Surgical photographs	461,000	473,000		Institutional	
4	medical_cases20	21/07/2016	352	Arabia	Medicine and Health	Medical quizzes, surgical images, case studies, memes, informational medical diagrams	55,600	67,200	Commercial	Individual	Student
4	study_4_medicine	28/07/2016	227	England	Medical School, Medical Mnemonics and MCQs	Informational diagrams, Annotated radiographs, dissection specimens	4081	4146	Profit making	Individual	Student?
4	medicinaclinica	06/04/2017	758	Hospital Medicine		Surgical photos and videos, inspirational images, educational/interesting images related to medicine	762,000	804,000			
4	sprezyna92	17/08/2014	468	Poland	Medicine, Sport and Health	Self-promotion, medical notes, promotion of books and films, medical motivation	11,500	14,000	Profit making	Individual	Student
4	injectiondaily	29/06/2016	177		Medical Facts	Fact images related to medical practice, disease and the body	82,200	98,400			
4	accademyofsurgeons	13/01/2017	145		Surgery and Medicine	Surgical images with questions and answers in the description, memes, jokes, rare surgical cases	116,000	150,000		Institutional	
4	talkinmedicine	31/05/2017	217	USA	Medical and Health	Photos/videos of rare medical cases with description headlines	35,400	Gone		Institutional	
4	surviving.medicine (Medical Motivation)	07/03/2017	269	USA	Medical Motivation, Podcast promotion	Medical photographs and illustrations with motivational taglines and quotes	12,300	16,200	Profit making	Institutional	Professionals
4	medplay.co.uk (Medicine MCQs - MedPlay Ltd)	12/03/2017	272		MCQ’s for Medicine, Nursing, Pharmacy and Premed students	Multipl-choice questions relating to anatomy, disease, diagnoses, physiology	12,800	30,200	Profit making	Institutional	Education company
4	medical_blog_	05/03/2017	689		Surgeries and Clinical Cases	Surgical images and videos	11,300	gone		Institutional	
4	academyofsurgery	10/10/2017	19		Medicine and Surgery	Surgical images and videos	3275	6032			
4	medbases	18/07/2017	218		Medicine and Surgery	Surgical images and videos, radiograph images, annotated specimens	17,000	Gone - replaced			
5	dentistrymyworld	06/03/2015	1841		Dentistry	Dental images and videos, rare cases, cosmetic dentistry photographs, memes, radiographs, dental equipment, seasonal posts product advertisement	332,000	355,000	Profit making	Institutional	
5	dentistrymyworld1	29/12/2015	3,311		Dentistry, Health and Wellness	Animated videos of dental procedures, case study videos, mems, dental-related photographs	873,000	124,000	Commercial	Institutional	
5	dentistryxworld	06/07/2017	10		Dentistry	Dental procedures, techniques, head anatomy	4181	3907		Institutional	
5	dentistry.world	24/08/2015	2438		Dentistry	Case study videos, procedure videos, orthodontics, before and after images, product advertising, information images	277,000	323,000	Profit making	Community	
5	dentistryzone	20/12/2016	743	Worldwide	Dentistry and Dentists	Videos and photos of rare cases, before and after photos, anatomy diagrams, cosmetic dentistry	16,100	33,100			
5	dentistry_forum	25/09/2014	1720	Worldwide	Dentistry education	Before and after treatment photos, radiographs, treatment videos	255,000	279,000			
5	identistry	02/07/2015	4942		Dentistry	Clinical videos and photos, related photos, comics, jokes, memes	282,000	334,000		Individual	
5	dentistrypage	29/03/2017	365		Dentistry	Before and after treatment photos, cosmetic dentistry, dental advertisements, related photos	64,200	62,500	Profit making		
5	dentistry corner	26/03/2016	1034		Dental-related content	Clinical cases, memes, jokes, related to dentistry photos, anatomy, reposted content	22,600	24,500	Profit making		Website
5	dentistryglobal	11/12/2016	564	Worldwide	Dentistry	Educational videos, comics, puns, anatomy, tooth-related merchandise	71,600	77,500		Community	
5	dentistryscience	04/12/2016	749		Dentistry for professionals	Teeth, before and after treatment photos, educational videos, memes, tooth anatomy	62,400	98,300		Community	
5	dentistrysc	06/04/2017	464	Worldwide	Dentistry	Dental cases, anatomy, head and neck diseases,	19,400	33,800		Institutional	Community
5	dentistry2day	26/12/2015	604	Worldwide	Dentistry	Clinical cases, dentistry in the media, memes,	78,100	82,000		Community	
5	dentists.doctors	22/12/2014	1003		Dentistry, Medicine, Pharmacy	Dental surgery, dental tools, anatomy, medical jokes, jokes, memes, related content	161,000	174,000			
5	dentistry_room	08/05/2016	292	Dubai/UAE	Cosmetic Dentistry	Photographs of teeth including before and after, poor teeth hygiene and prepped for treatment	7785	11,700			
5	dentistry.blog	27/05/2017	331		Orthodontics, cosmetic dentistry, dental surgery	Before and after photos, braces, oral surgery, jaw re-alignment, dental techniques,	9155	10,000		Individual	
5	dentistrymentor	26/03/2017	1106		Medical and Health, Dentistry	Teeth, before and after treatment photos, orthodontics, moulds, implants	6691	12,400			
5	dentistry_aesthetics	24/09/2017	235		Dentistry	Reposts of before and after treatment photos with descriptions of the treatment	5790	11,500		Individual	Student
6	dentistryeducation	14/10/2017	6		Dental Education	Tooth anatomy, head and neck anatomy	1006	2331			
6	guided.dental	13/08/2017	82		Dental Education	Oral surgery, anatomy, perio, pros/restorative, ortho, endo, implantology - quiz photos with answers in the next post	13,400	21,900			
7	about_dentistry	07/08/2015	2170	USA	Dental Education	Videos of treatment, memes, related content, merchandise advertisement	302,000	335,000	Profit making		
7	amelia_studies	19/03/2016	284		Medical Student Notes	Photos of medical notes containing information	89,300	97,300			
7	brainyleslie_	20/06/2017	143	USA	Neurosurgery	Brain scans of clinical cases, personal stories, medical advice, team photos	1039	3407		Individual	Staff
7	neuroradcases	22/08/2017	78		Neuroradiology	Radiographs of clinical cases relating to the spine, brain and skull	1235	2123		Individual	Staff
7	blu_med	27/09/2016	76		Medical and Health	Medical photos with educational captions, radiographs, summaries of diseases	513	707		Individual	
8	arorameded	05/07/2016	1274		Global Medical Education	Information sheets on medical topics, selfies, memes	1344	Gone		Individual	Professional
9	sarahjclifford	14/03/2014	363	England	Medical Illustration	Hand-drawn notes, medical drawings, selfies, promotions	73,600	75,900	Profit making	Individual	
9	mike.natter	21/12/2014	1174	USA	Medical drawings	Medical illustrations and diagrams, humorous cartoons, practicing doctor advice	74,900	77,900	Profit making	Individual	
9	thegirlwiththewhitecoat	03/01/2018	12		Medical Notes	Hand-drawn notes, medical inspiration	466	1877		Individual	
9	the_littlemedic	08/04/2017	69		Revision Notes	Hand-drawn medical school notes	2519	3764		Individual	
9	simplify.drugs	15/09/2016	420		Pharmacology Education	Memes, MCQs, information on medical diseases, related content	53,300	67,700			
9	geekymedics	10/01/2017	57		Medical Education	Medical diagrams, anatomy, medical technique videos, cranial nerve education	11,300	13,500		Website	
9	thexraydoctor	31/01/2015	1401		Radiology	Radiographs of clinical cases in medicine	32,400	37,000		Individual	
9	med_life	19/09/2016	310		Medical Education	Information sheets of medical cases and anatomy and physiology	90,200	10,7000	Profit making		
9	myneurosurgery	03/08/2013	848		Neurosurgery Education	Neurosurgery clinical case photographs, radiographs of the head and spine	105,000	109,000			
10	medicaltalks	20/04/2014	1465		Medical Case Studies	Photographs and videos of medical cases with medical descriptions	190,0000	210,0000		Community	
10	medicalpedia	05/06/2016	350		Medical Education	Rare or shocking medical cases with educational descriptions	236,000	371,000		Institutional	
10	medical_notes12	11/11/2016	3183		Medical Notes	Anatomical diagrams, MCQs inspirational quotes/pictures, clinical cases, information sheets, symptoms and diagnostic tools	169,000	228,000			
10	medicosity	19/05/2016	492		Medical Education	Clinical cases, memes, cartoons, related content,	45,500	61,700		Community	
10	medgeeksinc	10/08/2015	1711		Medical Education	Medical facts, information sheets on medicine and diagnosis, memes, MCQs, jokes, related content	200,000	216,000		Website	
10	remains2beseen	18/06/2015	1049		Pathology and Human Remains	Anatomy, post mortem practices, body parts, pathology, dissections	85,300	89,400		Individual	
10	anakkedokteran	14/05/2015	1085	Philippines	Medical Education	Information sheets/MCQs on medicine, medical news, inspirational posts	35,900	34,900		Individual	
10	medicalmad	14/06/2016	503	German	Medial Pictures and Videos	Medical case photographs and radiographs	14,200	Gone		Individual	
10	codexanatomicus	16/02/2017	126		Medical Artwork	Anatomical diagrams	65,800	94,700	Profit making	Individual	
10	medicalterms	11/01/2016	710	Medical Terms and Education	Medical definitions, information sheets on anatomy and medicine		200,000	240,000	Profit making		
11	odontologiapreventiva	22/02/2014	294		Medical and Health	Tooth anatomy, before and after teeth treatment, tooth growth, tooth facts, cartoons, orthodontic treatment information	72,000	86,100			
11	oidentistas	12/01/2018	57		Orthodontics	Before and after orthodontic treatment rare cases with explanatory descriptions, questions on cases with answers in the description/comments, memes, tooth factors, adverts	188,000	200,000	Profit making	Magazine	
12	anatomianatomy	16/11/2014	1289		Muscles, Bones and Joints	Anatomy of the human body, relevance to exercise, CNS	86,000	104,000			
12	anatomiae	27/05/2015	799		Anatomy Education	Anatomy, medical cases, adverts, cartoons, anatomy-related artwork	600,000	670,000		Website	
12	_anatomia_	11/11/2016	608	Swizerland	Anatomy	Anatomy, neurosurgery cases, bacteria, medical-related content, memes	44,400	568,000	Profit making	Institutional	
13	dr.daniel.fortino	21/12/2017	30	USA	Implant and Periodontal Surgery	Clinical case photographs and radiographs, photos of the dental team, before and after treatment photographs	557	1240		Individual	
13	dental_anatomy	17/09/2014	456		Dental Anatomy	Head and Neck anatomy, anatomy on other body systems, models for practical anatomy	764	814			
13	dr.smile	30/03/2014	1740		Dentistry	Tooth anatomy, clinical cases, related content, radiographs	326,000	344,000		Individual	

This audit revealed 80 Instagram accounts relevant to anatomy, particularly focussed on medicine and dentistry. Of the 80 accounts searched, 2 begun posting in 2018, 25 in 2017, 24 in 2016, 15 in 2015, 12 in 2014, and 2 in 2013. The number of posts per account had a range of 6–4942 and the number of followers between 513–2.1 million.

The majority of accounts did not specify a country of origin, those that did were mostly from the USA. The most common subject area was medical education. However, there was also a substantial number of accounts focussed on a single profession within medicine, such as neurosurgery or orthodontics. A common observation, especially amongst the most followed accounts, was the appearance of memes (a humorous, often relatable, photo with a bold caption) and cartoons, which were present in 26% of the accounts reported. Educational content was most commonly presented as a photo of a clinical case, often taken in operation theatres, with an associated description providing scientific information about the treatment and outcome of the patient. Those undertaking artwork as educational content only made up 5 of the 80 accounts.

Of all the accounts investigated, 42 focussed only on medical-related content, 23 on dental-related content, 6 on gross anatomy, and 9 were relevant to both medicine and dentistry. Within the dental field, there was frequent use of ‘before and after’ photos of patients’ teeth from a procedure. There were also many radiographs on different procedures and related dental humour. For medicine-focussed accounts, rare or shocking cases were common. Multiple-choice questions associated with uploads were also popular, with the answer to the question often presented in the description. Brain scans and photos of brain surgery are uploaded regularly.

None of the accounts explicitly stated that there was a procedure to ensure quality control over their content, so there was no guarantee that the information being spread was correct. Furthermore, whilst the majority of accounts did not use the platform to generate revenue, 22 of the accounts (27.5%) seemed to adopt some kind of advertising within their content. Owners of the Instagram accounts included both institutions and individuals. Some accounts identified whether students, professionals, or other individuals were posting, but not all specified.

## Discussion

The majority of anatomy-related Instagram accounts (see Table 1) first posted within the last 2 years, indicating how recent the growth of Instagram is as a platform for dental and medical education.

The main benefit of Instagram over other social media is that it focuses on photos and videos, which is valuable for the subject of anatomy as it relies heavily on visual resources to support learning. However, we acknowledge that two-dimensional photos may lack the capacity to develop necessary visuospatial skills to help formulate a three-dimensional understanding of anatomy, and so, in this respect, Instagram provides learning opportunities comparable to that of an anatomical atlas. However, the act of creating images is considered an educational process in itself [15], and the mobile accessibility of posts may support the timeliness of particular learning objectives for some groups of students. It also means that users from all around the world can gain an understanding of anatomy without requiring a detailed knowledge of a language.

Passive learning techniques (mentioned as a disadvantage to YouTube videos) is also a limitation applicable to Instagram, since it may provide students with inflated confidence in their knowledge and self-assessment ability [14]. Some individuals may use Instagram as a form of procrastination which may distract them from engaging with genuine educational posts. One approach used to encourage engagement is via the publication of images associated with engagement by the user such as multiple-choice questions with the answer revealed in the following post. Another method involves writing thought-provoking questions in the description to encourage interaction and discussion, as demonstrated by the account ‘medical_notes12’. Despite the relatively small number of Instagram accounts relating to medical and dental education, Instagram still offers some benefits over YouTube. For example, it limits the content to images and short videos, which may better suit some learning approaches.

The common use of memes, jokes, and cartoon references demonstrates the need for positive and light-hearted morale boosting content which is comparable to reports on how Twitter is utilised in education [5]. The success of each post is determined by interactions, discovery, reach, impressions, and the number of likes it receives, which is obtainable from the insights tab with each Instagram account. Our own module evaluations at the University of Southampton revealed that student’s preference was for colourful images with annotated/associated descriptive text over that of 60-s videos, summary tables, or labelled images. This finding is supported across Instagram education sites more broadly (see Table 1). It has been suggested that accounts created by students for students are more cognitively and socially congruent as they are relatable to peer-assisted learning benefits [16], potentially indicating that peer-related content is preferable to that of professional or institutional content [9]. This hypothesis would certainly align with the transition to Instagram for many younger social media users and with the suggestion that students, given the choice, are opting in favour of more traditional lines of communication and support from academic and clinical staff [9]

Keenan et al. describes the main barriers of social media in education and includes key aspects such as the need to have educational value, professionalism, staff knowledge and experience, staff motivation, and student usage [17]. These values and criteria are very much applicable to Instagram and would suggest that a successful educational account would require a committed person/team (preferably managed by a dedicated person) to source and post relevant content which is quality-controlled. Similar to other social media platforms, Instagram posts that are not made public will require students to have an Instagram account in order to access them, which poses an issue around accessibility. However, educators can also make the resources available elsewhere, such as via ‘widgets’ for Institutional learning management systems (such as Blackboard) that enable the account feeds to be embedded within web pages. This will ensure that all students have access to the content. The report by Keenan et al. also describes the value of social media sites in education more broadly. Themes such as encouraging engagement outside of the classroom, collaboration, and dissemination are all important considerations—plus the familiarity and immediacy of content were shared as positive ideas towards what sites like Instagram could bring to teaching [17].

By using our understanding of how social media use within education is evolving, medical and dental educators can utilise the ‘intuitive interface’ of Instagram to upload engaging information and optimise the delivery of content. They may be able to make innovative use of their content by adopting modern educational approaches in their teaching, such as ‘flipping the classroom’ or ‘blended learning’. However, considering the emphasis and popularity of informal peer-to-peer study-related uploads on Instagram, educators might want to think carefully about how they choose to engage their students using social media. There is most likely still room for professional, informative, and quality-assured profiles administered by academics. Particularly because so many young adults are shifting over to Instagram as their preferred social media platform [9].

As educational accounts continue to grow on Instagram, it will become increasingly important for the ethical considerations to be addressed. Several Instagram accounts upload operative procedures that include patients and/or cadaveric material, and the legislation surrounding this type of content will vary between countries. Although there are warnings over the display of sensitive material there are no details of informed consent from the patients or body donors. It will also be important for students to be aware of professionalism within their respected disciplines, particularly as it becomes more desirable to attract new followers with increasingly engaging content.

## Conclusion

The findings we report here suggests that Instagram could be well-suited to support dental and anatomy education, although there is a lack of performance data amongst the literature to allow for an endorsement of its true impact on education. Instagram shows many similarities to other big social media sites like Facebook, Twitter, and YouTube in how it is easily accessible through smartphones and computers—the main differences between them are what make them suited for different purposes and how they attract divergent audiences.

Facebook is mostly tailored towards enabling individuals to communicate with one another through ‘likes’ and comments on photos or ‘statuses’. However, issues like professionalism arise as an individual must interact on the site via their personal profile which can lead to privacy and safety concerns. YouTube’s content is unidirectional media that relies on the viewers’ concentration for the duration of the video and is less focussed on interaction or responses. Its major benefits centre around the ability to pause, rewind, and replay video content, and so could very useful, particularly for instructional educational videos or screencasting tutorials [18]; however, it is not tailored for photos or still images. The use of hashtags is popular across all social media sites but has a particular resonance with Instagram as a topic marker which enables content of interest to be searched for easily, a particularly useful feature for education.

In light of the aforementioned limitations of Instagram, such as the lack of quality control and patient confidentiality, it is clear that there is scope for modifications to its terms and conditions and regulatory procedures that would make it more suitable for medical and dental education. Examples of improvements include the verification of quality accounts, which could be achieved by having educators’ peer-reviewing relevant accounts (such as those provided by our audit) and approving content. Evidence of consent is another issue which could be acted upon through internal processes. One suggestion would be for posts to be submitted to a review process before they eventually go live - if social media can implement this system for paid promotions and marketing, then it would stand to reason that they can also administer it for ethical purposes.

Given the diversity of profiles amongst staff and students, one possible approach to building successful Instagram profiles might be through co-creation. This would potentially witness the advantages of creative motivational content from students, integrated with professional quality standards offered by staff. Working in partnership with students is gaining significant traction in higher education since it was endorsed by The Higher Education Academy as a form of best practice [15]. In addition, on applications like Instagram, it may be more cost and time efficient for students to create the content as a way of providing them with opportunities to build their knowledge through the design process.

In the current age of advancing technology, the future role of Instagram in the technological sphere remains to be seen. However, at present, it is a potential tool which can complement the student experience in a similar way to that of Facebook and YouTube; however, it cannot replace hands on anatomy education and its full impact on learning remains to be seen.
